# Visual Ecology and the Development of Visually Guided Behavior in the Cuttlefish

**DOI:** 10.3389/fphys.2017.00402

**Published:** 2017-06-13

**Authors:** Anne-Sophie Darmaillacq, Nawel Mezrai, Caitlin E. O'Brien, Ludovic Dickel

**Affiliations:** UMR Centre National de la Recherche Scientifique Université de Caen-Université de Rennes 1, Normandie Université, Université de Caen Normandie, Team NECCCaen, France

**Keywords:** cephalopod, vision, embryo, brain, polarization, camouflage, behavioral plasticity

## Abstract

Cuttlefish are highly visual animals, a fact reflected in the large size of their eyes and visual-processing centers of their brain. Adults detect their prey visually, navigate using visual cues such as landmarks or the *e*-vector of polarized light and display intense visual patterns during mating and agonistic encounters. Although much is known about the visual system in adult cuttlefish, few studies have investigated its development and that of visually-guided behavior in juveniles. This review summarizes the results of studies of visual development in embryos and young juveniles. The visual system is the last to develop, as in vertebrates, and is functional before hatching. Indeed, embryonic exposure to prey, shelters or complex background alters postembryonic behavior. Visual acuity and lateralization, and polarization sensitivity improve throughout the first months after hatching. The production of body patterning in juveniles is not the simple stimulus-response process commonly presented in the literature. Rather, it likely requires the complex integration of visual information, and is subject to inter-individual differences. Though the focus of this review is vision in cuttlefish, it is important to note that other senses, particularly sensitivity to vibration and to waterborne chemical signals, also play a role in behavior. Considering the multimodal sensory dimensions of natural stimuli and their integration and processing by individuals offer new exciting avenues of future inquiry.

## Introduction

**Figure 1:**
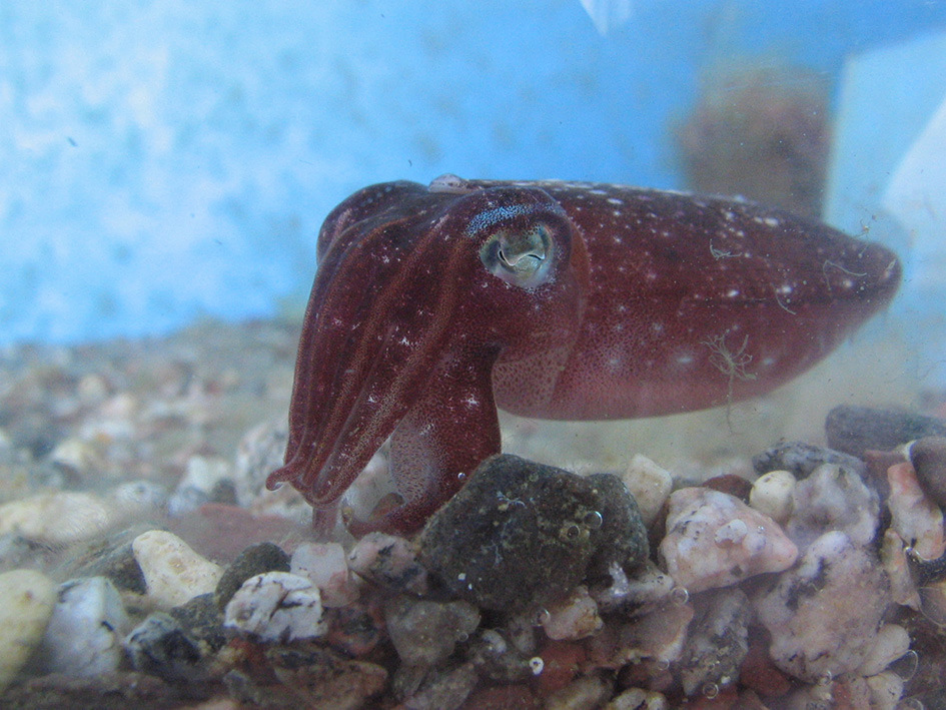
Eyes of the cuttlefish *Sepia elongata* caught off the coast of Eilat (Gulf of Aqaba, Israel; photo AS Darmaillacq).

**Figure 2:**
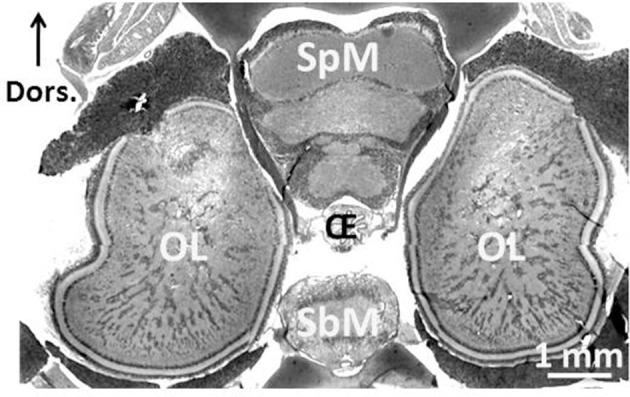
Central nervous system of 3-month-old *Sepia officinalis* cuttlefish. Frontal section. Prenant-Gabe trichrome stain. Abbreviations: OL, optic lobe; SpM, supra-esophageal mass; SbM, sub-esophageal mass; Oe, esophagus. Modified from Jozet-Alves et al. (2012a).

One of the most remarkable experiences one can have as a SCUBA diver is an encounter with a cuttlefish. Not only is it unexpected (during daytime, cuttlefish are mostly camouflaged, and only an experienced eye is likely to spot one), but you have a strange feeling of being observed! Indeed, the eyes of the cuttlefish are large and captivating (Figure 1). They are single-chambered camera-type eyes whose structure strikingly resembles that of vertebrates. This convergence is unique among invertebrates and was probably driven by shared ecology and competition with fish (Packard, 1972). Another indication of the importance of vision to cuttlefish, though other senses are important, is the size of the optic lobes. These two bean-shaped lateral nervous structures process visual information and occupy 140% of the whole central nervous system (Nixon and Young, 2003; Figure 2). The primary purpose of the visual system is to recognize objects so that individuals may interact with them appropriately and execute the behaviors necessary for survival. Vision plays a crucial role in the early life stages, as functional vision is essential for perception of prey, predator avoidance and visually-guided behavior (e.g., predation, Darmaillacq et al., 2004; camouflage, Zylinski et al., 2012; navigation, Cartron et al., 2012). Consequently, the early development of functional vision is critical because it enhances the chances of survival. Although the visual capacities of cephalopods have been studied extensively in adults, few studies have investigated their development. Indeed, embryos were traditionally considered to possess only limited abilities because of the immaturity of their developing brains. In this review, we will describe how the visual system develops in embryos and how it allows embryonic visual learning. We will also summarize our knowledge of some of the interesting particularities of cephalopods: polarization sensitivity (PS) and contrast perception (Shashar et al., 2002), and that of visual lateralization. Lastly, more recent data regarding the development and plasticity of defensive behavior in juveniles will be presented.

## Embryonic development of the visual system and embryos' responses to visual stimuli

### Development of sensory systems

*Sepia officinalis* eggs are laid in clusters on various kinds of rigid support such as algae, tubeworms, ropes or nets. Unlike other species of *Sepia*, the eggs are usually darkened with maternal ink but become more translucent due to the expansion of the capsule during embryonic development (Boletzky, 2003). *S. pharaonis* eggs are completely translucent.

During the final phase of embryonic development (stages 23–30; Boletzky et al., 2016), rhythmic mantle contractions are visible through the egg capsule after removal of the outer darker envelopes. These can be measured to assess embryonic responses to various external stimuli. Like this, Romagny et al. (2012) showed that in cuttlefish embryos, the order of the onset of function of chemosensitivity, touch and vision follows the same sequence as that of birds and mammals, with the visual system being the last to develop. Neurobiological data illustrating the early development of sensory neurons in embryos support these behavioral observations (Baratte and Bonnaud, 2009). This is another evidence of convergent evolution between cephalopods and vertebrates, perhaps instigated by similar environmental pressures and direct competition (Packard, 1972). Because embryonic development takes place outside of the mother and in the absence of direct parental care, there is strong evolutionary pressure for the rapid development of functional sensory systems, so that predators can be avoided and feeding can begin. Unlike some vertebrate species, in which the visual system is still immature at birth (Bremner et al., 2012), indirect evidence suggests that cuttlefish embryos can discriminate objects outside the egg. However, to date, no systematic study has been conducted on the development of retina morphology and physiology in the embryo (but see Imarazene et al., in press).

### Embryonic visual responses

There is increasing empirical evidence that prenatal experience influences postnatal perception, cognitive performance and behavior. Embryonic perceptual learning, (tested in neonates) has been demonstrated across many taxa, including insects (Caubet et al., 1992), amphibians (Mathis et al., 2008), rats (Hepper, 1988), dogs (Wells and Hepper, 2006), precocial birds (Sneddon et al., 1998), altricial birds (Colombelli-Négrel et al., 2012, 2014), and humans (Moon et al., 2013).

**Figure 3:**
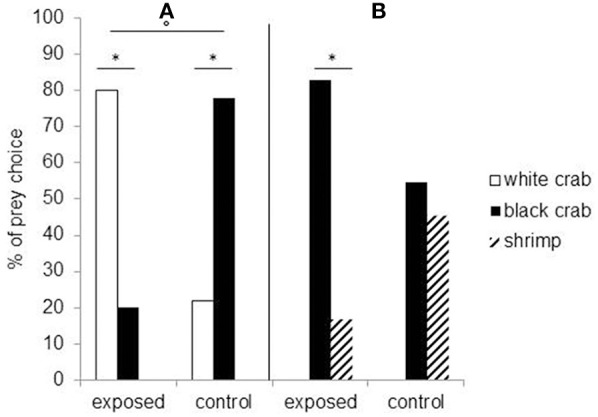
Seven-day-old cuttlefish's prey choice depending on whether they have been exposed to white crabs during embryonic development (“exposed”) or not (“control”). **(A)** To the left of the vertical: when they are presented a choice between white and black crabs. **(B)** To the right: when they have a choice between black crabs and shrimp. ^*^Significant prey preference within groups (chi-square exact test: *p* < 0.05) and °significant difference in prey choice between groups (Fisher's exact test: *P* < 0.05). Modified from Guibé et al. (2012).

Studies showed that embryonic visual experience affects both feeding and defensive behaviors. Cuttlefish embryos visually exposed to juvenile crabs for the last week before hatching will prefer crabs to their innately preferred shrimp prey (Darmaillacq et al., 2008). Likewise, cuttlefish innately prefer black crabs to white crabs but will preferentially select white crabs following embryonic exposure to them (Guibé et al., 2012; Figure 3A). Thus, it seems that not only do the cuttlefish pay attention to the shape of the prey (crab vs. shrimp) but also to its brightness. The relative importance of shape and brightness can be inferred from the fact that cuttlefish select black crabs over shrimp after embryonic exposure to white crabs, suggesting that they are generalizing the characteristics of a learned preference (crab shape) to the closest alternative (black crab) if the preferred item is not present (Guibé et al., 2012; Figure 3B).

Juvenile cuttlefish, that spontaneously prefer dark shelters, lose this bias when they have been exposed embryonically to white ones (Guibé and Dickel, 2011). Lee et al. (2012) also showed that cuttlefish raised prenatally in a visually enriched have a preference for high-contrast backgrounds whereas control cuttlefish have no substrate preference. More experiments are needed to study the direct response of the embryo to visual stimuli and the development of related brain structures.

These preferences for certain visual characteristics such as shape and brightness following embryonic exposure are relatively straight-forward. In contrast, chemical exposure to waterborne cues from shrimp or crab alters visual preferences after hatching in a less explicable fashion. Embryonic exposure to crab odor and blank seawater had no effect on the normal preference for shrimp; exposure to shrimp cue however resulted in a reversal of the normal shrimp preference (Guibé et al., 2010). The authors suggested that this is possibly due to cross-modal effects, in which odor cue modulates a primarily-visual preference. Alternatively, it could be that because embryos in this experiment were exposed to the odors of adult shrimp and crabs and they were somehow able to determine the size of the animal by its odor cue, perceiving them as a danger rather than as prey. Repeating these experiments with shrimps and crabs of various sizes could determine whether age causes differences in odor cues that are distinguished by cuttlefish.

## Development of PS, contrast sensitivity, visual acuity and visual lateralization

The cephalopod rhabdomeric-type eye has only one type of photoreceptor. The microvilli of neighboring photoreceptors are arranged orthogonally in the retina which confers sensitivity to the linear polarization of light (Shashar et al., 2002), one of the main properties of light in shallow water (Cronin and Shashar, 2001). Cephalopod eyes are positioned laterally on the head allowing both a monocular and a binocular vision.

### Spatial resolution and polarization sensitivity

Spatial resolution (or visual acuity), is the ability to discriminate fine detail (Tansley, 1965), and plays an extremely important role in the lives of animals, as it allows them to navigate in space, evade predators, catch prey, and in some species differentiate between males and females. Using an optomotor apparatus and stripes of different width, Groeger et al. (2005) showed that visual acuity improves as cuttlefish grow, ranging from a minimum separable angle of 2.5–0.57° (a decrease in this angle value means a better spatial resolution). A decrease in light intensity affects visual acuity whatever the age of the individual.

**Figure 4:**
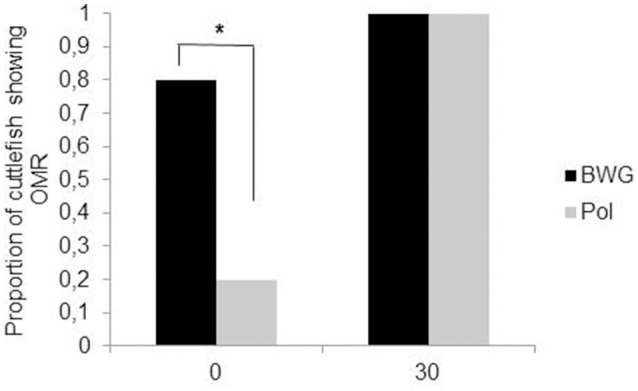
Proportion of the cuttlefish (*N* = 10 per group) that showed an optomotor response (OMR) to BWG (luminance only; black) or Pol (polarization; gray) patterns rotating at a velocity of 30 deg s^−1^, at hatching (0) and at the age of 30 days. Asterisks indicate a significant difference in the percentage of cuttlefish showing an OMR between the BWG and Pol patterns McNemar's test, (*P* < 0.05). Modified from Cartron et al. (2013a).

Polarization sensitivity (PS) improves the visibility of objects by enhancing the contrast between them and the background. In cephalopods, PS increases the success of predation on transparent prey or silvery fish (Shashar et al., 1998, 2000); in cuttlefish, it may also play a role in communication between adults (Shashar et al., 1996; Boal et al., 2004) and in navigation (Cartron et al., 2012). PS matures gradually after hatching. Cartron et al. (2013a) found that only 20% of cuttlefish hatchlings showed an OMR to a polarized striped pattern when it was rotated slowly. The proportion of cuttlefish responding increased throughout the first month of life (100% by the age of 30 days; Figure 4). However, a choice test with fully polarized or depolarized mysids (transparent shrimps) showed that 1 week-old cuttlefish detect polarized shrimp faster than non-polarized, suggesting an earlier maturation of PS (Cartron et al., 2013a). These apparently contradictory results could be explained by the motion of the rotating pattern in the OMR apparatus compared with the more stationary prey. It is possible that polarization contrast is more useful in assessing the shape of prey and that motion can interfere somewhat with this ability. This deficiency could be mitigated by the fact that polarization is not the only quality of light to which cuttlefish are sensitive. Though colorblind (Mäthger et al., 2006; but see Stubbs and Stubbs, 2016), cuttlefish are sensitive to contrast. Indeed, most hatchling cuttlefish (75%) showed an OMR to the black, white and gray striped pattern rotating at the lowest velocity, with the proportion reaching 100% by the age of 1 month. Thus, it can be hypothesized that polarization and luminance signals are processed separately and may play different roles in vision as observed in insects (Pfeiffer et al., 2005). In the desert locust *Schistocerca gregaria* for instance, a group of neurons in the central complex (a neuropil in the center of the brain), has been found to be sensitive to polarized light while neighboring neurons are not (although all neurons responded to unpolarized light). More experiments, notably electrophysiological and immunochemistry investigations, are needed in order to determine the neural pathways for polarization and luminance information processing in cuttlefish.

### Ontogenesis of visual lateralization

Cerebral lateralization, a trait that is widespread in animal kingdom (Vallortigara and Rogers, 2005; Frasnelli et al., 2012), is often revealed behaviorally by motor and perceptual asymmetries. In cuttlefish, adults have a preference for turning right or left (side-turning preference) in a T-maze (Alves et al., 2007), which can be the result of an eye use preference as in octopus (Byrne et al., 2002, 2004). In juveniles, Jozet-Alves et al. (2012b) showed that although cuttlefish do not show any side-turning preference in a basic T-maze, they do develop a left-turning bias when shelters are available at the end of the maze's arms from the age of 3 to 60 days. Interestingly, when cuttlefish have been exposed to a predator odor before hatching, they preferentially turn to the left in the simple T-maze (Jozet-Alves and Hebert, 2013); this suggests an influence of environmental factors on the ontogenesis of visual lateralization in cuttlefish. This may be adaptive for young cuttlefish to decide rapidly which shelter to choose specially in a risky situation where predators are potentially present around.

### Influence of environmental constraints on PS and visual lateralization

*S. officinalis*, the European cuttlefish, is widespread in the English Channel, the Atlantic Ocean and the Mediterranean Sea where the turbidity can be high. On the other hand, *S. pharaonis* and *S. prashadi* are found in the Red Sea, on coral reefs, where the water is clearer. All these species are able to detect a polarized stimulus at higher turbidity levels than an unpolarized one (Cartron et al., 2013b,c), indicating that PS can improve the capacity for object detection through turbid waters when intensity information alone is insufficient. *S. officinalis* can detect objects, whether polarized or unpolarized, at higher turbidity levels than the other two (Cartron et al., 2013b). It is thus likely that PS, which is present in most cuttlefish species (but see Darmaillacq and Shashar, 2008), is a product of natural selection driven by visual features of the species' environment. This hypothesis is supported by the fact that the *S. officinalis* used in this experiment were lab-reared individuals that had never encountered turbidity, yet were still better-equipped to discriminate objects under these conditions.

## Defensive behavior

Cephalopods are known for their skills in quickly changing skin patterns in response to environmental change, a property referred to as “dynamic camouflage” (Hanlon and Messenger, 1996; Hanlon, 2007). This dramatic behavior is made possible by their unique skin structure that comprises three layers of cells: the chromatophores (containing dark-brown, reddish-orange or yellow pigments), within the most superficial dermis of the dorsal part of the mantle and arms, under the direct control of the brain; the iridophores, underneath, that reflect environmental light to create iridescence (particularly prominent on the ventral part); and the leucophores, the deepest, that reflect mainly white. Together with textural, postural and locomotor components, these chromatic elements constitute the “body pattern” of cuttlefish (Hanlon and Messenger, 1988). Body patterns displayed in a chronic fashion are mainly used for crypsis in juveniles as a primary defense strategy to avoid detection. Cuttlefish adopt a brightness similar to the substrate (general color resemblance), or a display disruptive colorations that breaks up the outline of the body so that the overall form of the animal is lost (Hanlon et al., 2009). The disruptive pattern has been the most studied. In the lab, it has been shown that artificial backgrounds such as 2d checkerboards can elicit this pattern (Chiao and Hanlon, 2001; Chiao et al., 2007). More, several authors (Chiao and Hanlon, 2001; Barbosa et al., 2007, 2008) showed that both check size and achromatic contrast affected the body patterns. Other characteristics of the objects present in the vicinity of cuttlefish are taken into account by juveniles such as the presence of egdes, the spatial phase and the three dimensionality (Chiao et al., 2005; Zylinski et al., 2009; Ulmer et al., 2013).

**Figure 5:**
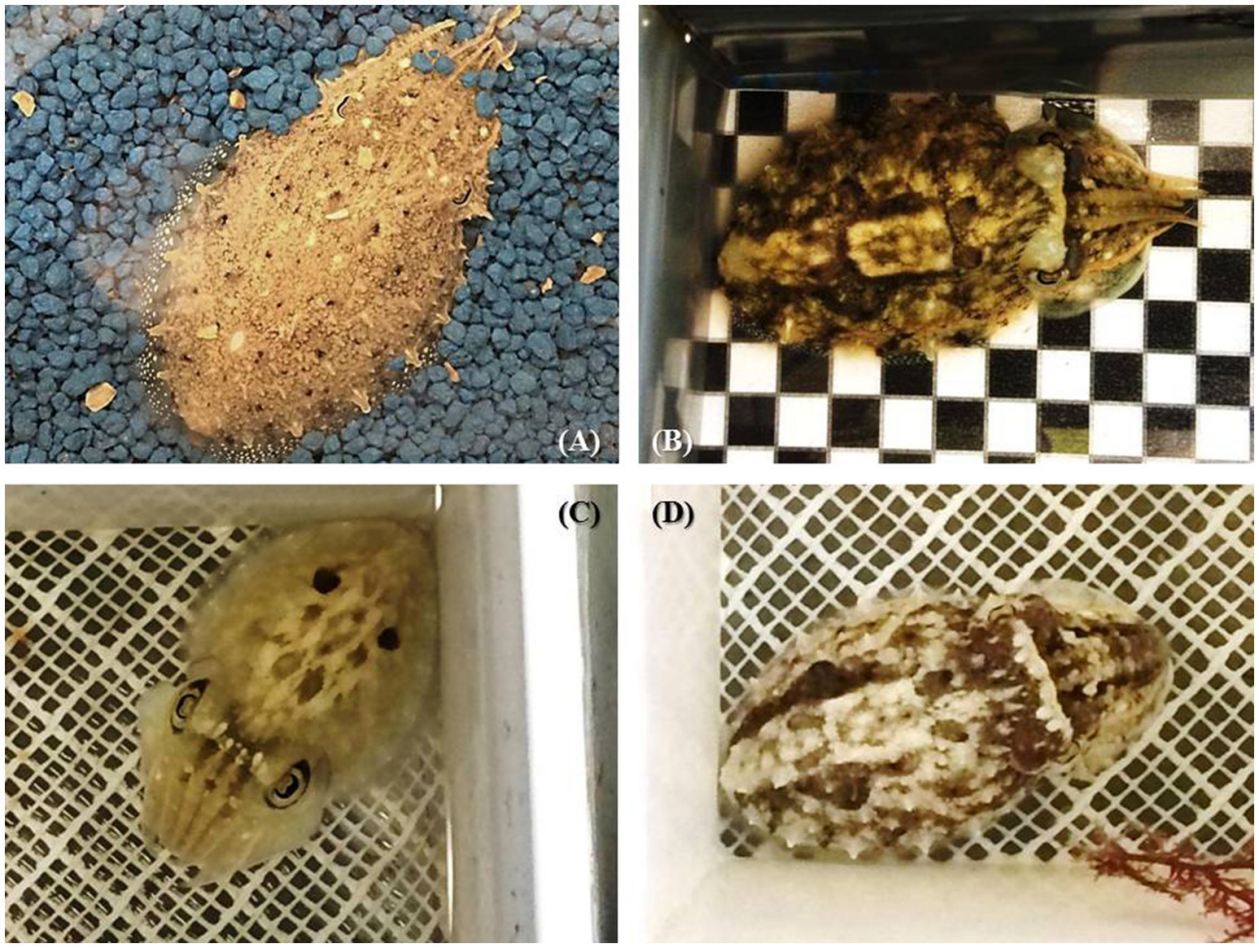
The diversity of body patterns displayed by 2-month-old cuttlefish (ca. 3–4 cm dorsal mantle length). **(A)** stipple-uniform pattern elicited on uniform blue gravel; **(B)** disruptive pattern elicited on a black and white checkerboard combined with mottle pattern; **(C)** deimatic pattern following exposure to a “threat” **(D)** mottle coloration with some components of the disruptive pattern (i.e., white square, white head bar, and paired black dots). Note that patterns are not always fully expressed but exist in combination with others and may or may not directly reflect the visual background.

Other body patterns (such as the deimatic and flamboyant displays) are shown in a more acute manner (only for a few seconds) and are used mainly as “secondary” defense strategies after a cuttlefish has been detected. Cuttlefish can also adopt a deceptive resemblance to natural objects in the environment (e.g., floating algae) to deceive potential predators or prey. In juvenile cuttlefish, uniform and mottle patterning are generally displayed on uniform/fine sandy backgrounds (Figure 5A) while disruptive coloration occurs on more patchy/contrasted substrates (Figures 5B,D). Uniform, mottle and disruptive patterns are usually mixed to varying degrees (Hanlon et al., 2009; Figures 5B,C,D), making camouflage “efficiency” very difficult to define or measure (see discussion in Hanlon et al., 2009). Last, in adults, body patterning plays a large role in intra-specific signaling, especially in agonistic and courtship behavior (Hanlon and Messenger, 1988). While social interaction between hatchlings appears to be non-existent (see Holmes, 1940; Hanlon and Messenger, 1996), it is still possible that body patterning also plays a role in signaling between young cuttlefish. This remains unclear as inter-individual communication has never carefully investigated in juvenile cuttlefish, and scarcely even in adults (see Boal et al., 2004).

Functional chromatophores first appear *in ovo* during stage 25 of embryonic development, when the dorsal mantle length of the animal is about 2 mm (Bonnaud-Ponticelli and Boletzky, 2016). While the total number of chromatophores increases with age, their density progressively decreases from 400 to 500/mm^2^ at hatching to 35 to 50/mm^2^ in adults (Hanlon and Messenger, 1988). Nevertheless, both juveniles and adults possess a high density of cells that allow them to express an infinite range of gradations of various components of their body patterns, depending on background and lighting (Hanlon and Messenger, 1988). Thirteen “typical” body patterns have been identified in adults, but since the body patterning related to sexual behavior is absent in juveniles, the number of color, postural-kinetic, and structural components is lower—only nine distinct patterns (Hanlon and Messenger, 1988). Qualitative changes in body patterning also occur in juveniles. For example, when a late juvenile (about > 6 weeks) or adult is threatened by a small predator, it often displays a “deimatic pattern” in an attempt at intimidation: it flattens its body and flashes two big spots against a white dorsal mantle in a manner resembling eyes (Figure 5C). In younger animals, this pattern appears very rarely (Thorpe, 1963; Hanlon and Messenger, 1988), and though the postural components are the same as in adults they flash not two but six dark spots (Hanlon and Messenger, 1988; Mangold, 1989) until about 2 weeks of age. While this version of the deimatic display is used sometimes, newly-hatched cuttlefish are more likely to respond to potential danger with a general darkening or blanching of its body or a cryptic flamboyant display (Hanlon and Messenger, 1988).

One wonders whether body patterning development in juvenile cuttlefish is rigidly fixed or is more influenced by prior individual experience. Simple observations of body patterning in early juveniles speak to this question: when placed on the same background different individuals display different body patterns, suggesting that the response is partially determined by previous experience. Other anecdotal and experimental evidence has the opposite implication however. Hanlon and Messenger (1988) released young cuttlefish (from < 1 to 17 weeks of age) previously reared in captivity into the field and observed that they concealed themselves effectively against every substrate encountered and were extremely difficult to see by human observers. Unfortunately, the personal histories of individuals were not described (i.e., whether they were reared in groups or in isolation, the amount of time spent in the wild before the behavioral observations, etc.), so we cannot make any definitive conclusions. Still, this observation suggests that body patterning development could be hard-wired since the impoverished artificial conditions of rearing do not seem to have any deleterious effects on the concealment skills in juveniles.

More controlled experiments also support an innate origin. Cuttlefish were reared in either “impoverished” conditions (housed individual tanks on a dark uniform background) or in “enriched” conditions (housed in groups in a variegated environment with sand, stones, shells, and artificial seaweeds) for 2 months (Poirier et al., 2005). Later, individuals from each group were tested on either a uniform gray substrate or checkered black and white background. In juveniles, a uniform background should elicit a uniform or slightly mottled body pattern (but see discussion in Hanlon et al., 2009), while a disruptive color pattern seems most adaptive against a contrasted background. The authors then assessed camouflage efficiency of by measuring the hue and intensity of various components of body patterning, on both uniform and contrasted substrates. At hatching, many cuttlefish display disruptive patterning regardless of background type. But starting at 15 days of age, cuttlefish previously reared in enriched conditions were better able to match both background types. Cuttlefish raised in enriched conditions also had greater cell proliferation in the optic lobes than those of cuttlefish from impoverished conditions. This makes sense, as the optic lobes are key structures controlling body patterning in cephalopods (Nixon and Young, 2003). Further evidence for greater innate or “hard-wired” control of body patterning comes from experiments with potential predators, in which *S. officinalis* was found to show the deimatic pattern toward small, low-threat teleost fish but not toward larger more dangerous predators such as sea bass or small sharks (Langridge et al., 2007; Langridge, 2009). Moreover, these reactions occur the first time such threats are encountered, suggesting innate recognition of threat type.

**Figure 6:**
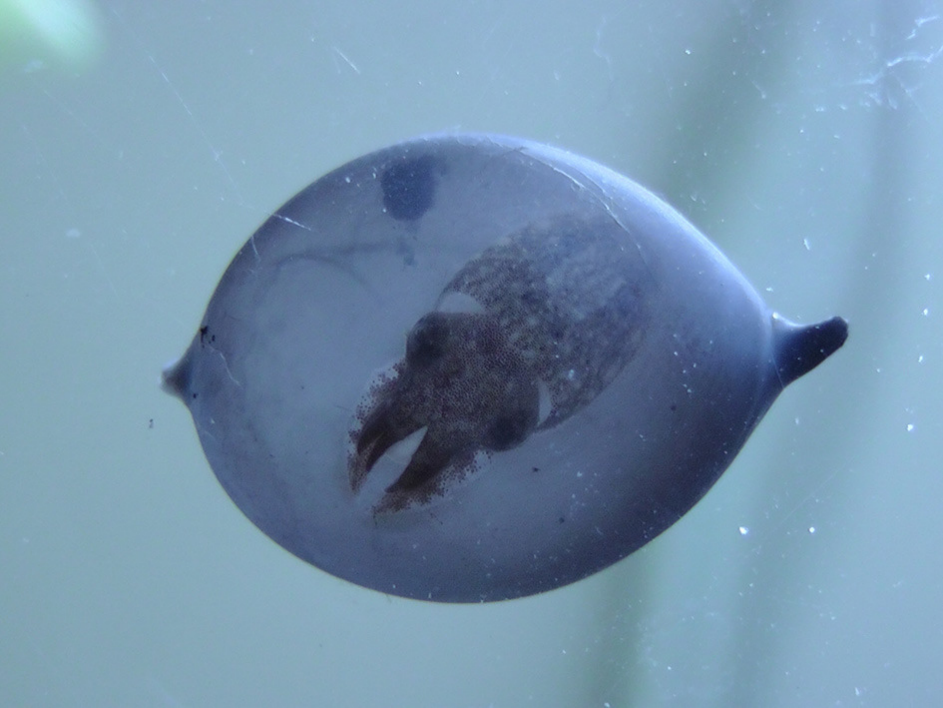
Stage 30 embryo (less than 1 cm) showing a mottle-disruptive coloration inside the egg. It has also squirted ink; note the cloud of ink in the perivitellin fluid. Note that the embryo is seen from under through a peeled *S. officinalis* egg (photo C.E. O'Brien).

While the preponderance of evidence suggests that body patterning is preprogrammed the fact that different individuals may use a different concealment strategies when placed in the same environment (Poirier et al., 2004), suggest some amount of experience-dependence, potentially through learning and phenotypic plasticity, although we cannot rule out the possibility that these inter-individual differences are the result of genetic history or parental experience. These data lead us to conclude that body patterning in cuttlefish is definitely not a simple stimulus-response process, as it is commonly presented in the literature. It probably involves a complex integration of visual information, genetic history and individual experience (West-Eberhardt, 1989), possibly even before hatching (Figure 6). Thus, further investigation of body pattern development could lead to insight not only about camouflage and defense, but also to a better understanding of learning, plasticity, decision making and higher-order cognitive processes in cephalopods (Vitti, 2012; Skelhorn and Rowe, 2016).

## Conclusion: embryonic ecology

In this review, we discussed the fact that the visual system is functional well before hatching, as indicated by indirect evidence from embryonic visual learning. By stage 25, the embryo's eyes are mature enough to perceive light and also to discriminate stimulus shape, movement and brightness. Unfortunately, little is known about the direct response of embryos to such stimulations and about the development of the brain structures that process visual information in cuttlefish, namely the optic lobes. The fact that cuttlefish are able to attend to and learn from their biotic and abiotic environment during the final stages of their embryonic development from the relative safety of their egg suggests that prenatal learning plays a large facilitative role in finding food and shelter after hatching. This ability may also enable prenatal social learning. Eggs are laid in clusters, and as a consequence, embryos are likely to be able see each other during development. Social rearing conditions after birth are known to have strong effects on growth and memory (Dickel et al., 2000), so the possibility of prenatal effects exists. No studies have yet addressed this, and experiments to test the effect of embryonic development in isolation on postembryonic behavior are needed.

Many questions about the development of vision in cuttlefish remain to be explored. For instance, do females actively choose their egg-laying site in order to increase offspring learning and survival (i.e., non genetic maternal effects)? Cuttlefish reproduce only once in their lifetime and hence, have only a single opportunity to produce offspring. This, combined with the potential for juvenile behavior to be shaped by embryonic learning, implies that strong selection pressure (based on the presence of predators, shelters or prey for juveniles) is exerted on females' decision. Since it has long been assumed that invertebrate behaviors are mostly genetically programmed, attention should be paid to such previously-neglected effects.

This synthesis highlights the importance of vision in embryo and juvenile cuttlefish behaviors. However, like other animals, cuttlefish live in a multisensory world, and even if vision appears predominant, their behaviors may be influenced by other senses. In most animals, the senses are not equal in their ability to provide accurate information about the environment (Bremner et al., 2012). For example, in a turbid environment, relying only on vision may be risky, and other senses may play a greater role. Komak et al. (2005) have demonstrated that young cuttlefish are sensitive to local water movements thanks to specialized cells on the arms and the head that are analogous to the lateral lines of fish. Water movement detected by these cells could alert cuttlefish to the presence of prey or predators before it is possible to see them. The importance of particular senses may also vary throughout the life of an individual. In cuttlefish, given the opacity of the egg capsule, the sensory world of embryos is probably dominated by chemosensory information. This likely changes as soon as the cuttlefish leaves the egg. Assessing the relative importance of vision and its interactions with the other senses through multimodal perception in different situations and at different ages offers exciting new tracks of research such as prey and predator recognition through visual and/or chemical information.

## Author contributions

All authors read and approved this version of the ms; ASD, wrote the main part of the article; NM, wrote the section about embryonic responses; LD, wrote the section about body patterns; CEO, co-wrote the section about embryonic behavioral response and copy-edited the ms.

### Conflict of interest statement

The authors declare that the research was conducted in the absence of any commercial or financial relationships that could be construed as a potential conflict of interest.
